# The regulation of IMF deposition in pectoralis major of fast- and slow- growing chickens at hatching

**DOI:** 10.1186/s40104-017-0207-z

**Published:** 2017-10-01

**Authors:** Lu Liu, Huanxian Cui, Ruiqi Fu, Maiqing Zheng, Ranran Liu, Guiping Zhao, Jie Wen

**Affiliations:** 1 Institute of Animal Sciences, Chinese Academy of Agricultural Sciences, Beijing, 100193 China; 2 State Key Laboratory of Animal Nutrition, Beijing, 100193 China

**Keywords:** Chicken, Gene expression, Intramuscular fat deposition, Pathway, Pectoralis major, Yolk at birth

## Abstract

**Background:**

The lipid from egg yolk is largely consumed in supplying the energy for embryonic growth until hatching. The remaining lipid in the yolk sac is transported into the hatchling’s tissues. The gene expression profiles of fast- and slow-growing chickens, Arbor Acres (AA) and Beijing-You (BJY), were determined to identify global differentially expressed genes and enriched pathways related to lipid metabolism in the pectoralis major at hatching.

**Results:**

Between these two breeds, the absolute and weight-specific amounts of total yolk energy (TYE) and intramuscular fat (IMF) content in pectoralis major of fast-growing chickens were significantly higher (*P* < 0.01, *P* < 0.01, *P* < 0.05, respectively) than those of the slow-growing breed. IMF content and u-TYE were significantly related (*r* = 0.9047, *P* < 0.01). Microarray analysis revealed that gene transcripts related to lipogenesis, including *PPARG*, *RBP7*, *LPL*, *FABP4*, *THRSP*, *ACACA*, *ACSS*1, *DGAT2*, and *GK*, were significantly more abundant in breast muscle of fast-growing chickens than in slow-growing chickens. Conversely, the abundance of transcripts of genes involved in fatty acid degradation and glycometabolism, including *ACAT1*, *ACOX2*, *ACOX3*, *CPT1A*, *CPT2*, *DAK*, *APOO*, *FUT9*, *GCNT1*, and *B4GALT3*, was significantly lower in fast-growing chickens. The results further indicated that the PPAR signaling pathway was directly involved in fat deposition in pectoralis major, and other upstream pathways (Hedgehog, TGF-beta, and cytokine–cytokine receptor interaction signaling pathways) play roles in its regulation of the expression of related genes.

**Conclusions:**

Additional energy from the yolk sac is transported and deposited as IMF in the pectoralis major of chickens at hatching. Genes and pathways related to lipid metabolism (such as PPAR, Hedgehog, TGF-beta, and cytokine–cytokine receptor interaction signaling pathways) promote the deposition of IMF in the pectoralis major of fast-growing chickens compared with those that grow more slowly. These findings provide new insights into the molecular mechanisms underlying lipid metabolism and deposition in hatchling chickens.

**Electronic supplementary material:**

The online version of this article (10.1186/s40104-017-0207-z) contains supplementary material, which is available to authorized users.

## Background

Lipid is one of the main nutrients in chicken yolk, and has an important role in fueling the embryonic development of chickens [1, 2]. When the glucose is fully consumed, lipids in the yolk are used predominantly at early embryonic developmental stages [3], and more than 90% of the embryo’s energy comes from fatty acid oxidation [4]. Differences in the usage of lipids from the yolk sac influence the different growth patterns seen in chicken embryogenesis [5].

Two mechanisms exist for consuming lipids from the yolk sac: absorption through blood circulation or absorption through the small intestine. A total of 80% of the lipids in the yolk sac are consumed by embryo growth by the final week of the prehatching stage [4, 6], and the remainder is transported into the embryonic abdominal cavity and deposited into nearby tissues after hatching [7, 8]. This results in a rapid increase in the content of intramuscular fat (IMF), which coexists with muscle tissue in breast and thigh.

A few studies on chicken lipid metabolism before and after hatching have been reported [9, 10], but there is still a lack of systematic research on the molecular regulation of IMF deposition from the yolk sac at hatching. In this study, with the aim of identifying global differentially expressed genes (DEGs) and pathways related to lipid metabolism in chicken breast associated with fast- and slow-growing breeds at hatching, a comparative analysis of the gene expression levels between fast- (Arbor Acres, AA; a commercial fast-growing broiler) and slow-growing chickens (Beijing-You, BJY; a slow-growing Chinese breed) was performed using microarray technology.

## Methods

### Animals and sample collection

Six AA and six BJY chickens (half male and half female) on the day of hatching were used in this study. Individuals within each breed had the same genetic background. Animal experiments were approved by the Science Research Department (in charge of animal welfare issues) at the Institute of Animal Sciences, Chinese Academy of Agricultural Sciences (CAAS), Beijing, China.

After birds had been weighed and their live weight had been recorded, they were sacrificed and the pectoralis major and yolk sac were excised. The pectoralis major samples were stored at −80 °C or −20 °C for RNA isolation and the measurement of IMF content. The yolk sac samples were stored at −20 °C for the measurement of total yolk energy content (TYE).

### Measurement of biochemical indexes

TYE was determined with a Parr 1281 bomb calorimeter (Parr Instrument Co., Moline, IL, USA). IMF content in pectoralis major was measured by the Soxhlet method [11], using anhydrous ether as the solvent, and is expressed as a percentage of dry tissue weight.

### Total RNA preparation, microarray hybridization, and analysis of DEGs

Total RNA samples from six AA and six BJY chickens were isolated individually and pooled for microarray analysis with equal amounts (1 μg) from every sample. Microarray hybridization was performed by Shanghai Biotechnology Corporation (Shanghai, China) using an Agilent Chicken Gene microarray (ID: 015068). Array scanning and data extraction were accomplished following standard protocols. The normalized fluorescence intensity values from each dye-swapped experiment were averaged separately, after which averaged sample and reference fluorescence values were log_2_-transformed for each probe. The expression value of each probe set was normalized and calibrated using the RMA method. DEGs were screened and genes were considered to be differentially expressed only when the relative abundance fold change between the two breeds exceeded 2.

### Quantitative real-time PCR (qPCR)

Individual RNA samples from all chickens were used. All PCR primers were designed at or just outside exon/exon junctions to avoid the amplification of residual genomic DNA, and specificity was determined using BLASTN (Table 1).

**Table 1:** The specific primers for qPCR in this study

Gene	Sequence	Product size	Accession NO.
*THRSP*	F:5′-ATCAAGCCCGTGGTGGAGC-3′ R:5′-CTTTGGTGTTTTTGGTGAGGTCG-3’	184 bp	NM_213577
*ACACA*	F:5’-AACCTGCTAAACCCCTGG-3′ R:5′-AGTCCCAAATCCGAAAGG-3’	175 bp	NM_205505
*ACSS1*	F:5’-TGGGAGATGTTACCACAC-3′ R:5′-GCAGAATACACCAAGAGAG-3’	181 bp	XM_415011
*PPARG*	F:5’-TAAAGTCCTTCCCGCTGACCAAA-3′ R:5′-AAATTCTGTAATCTCCTGCACTGCCTC-3’	230 bp	NM_001001460
*LPL*	F: 5’-AGGAGAAGAGGCAGCAATA-3′ R:5′-AAAGCCAGCAGCAGATAAG-3’	222 bp	AB016987
*FABP4*	F:5’-GGGGTTTGCTACCAGGAAGATG-3′ R:5′-CATTCCACCAGCAGGTTCCC-3’	276 bp	NM_204290
*RBP7*	F: 5’-TTCCATCCATACCACAAGCACA-3′ R:5′-AGTGAGTCCAGCCCCTGTTCTT-3’	179 bp	XM_417606
*DGAT2*	F: 5’-AATGGGTCCTCACGTTCC-3′ R:5′-TGGTGGTCAGCAGGTTGT-3’	237 bp	XM_419374
*GK*	F: 5’-TATGGCTGCTACTTTGTGC-3′ R:5′-GTATCCCGCAGTCCTTGT-3’	187 bp	XM_416788
*ACAT1*	F: 5’-CTCCAGCAAGACAGGCAGT-3′ R:5′-CACCAGCAACCATTACATCC-3’	150 bp	XM_417162
*ACOX2*	F: 5’-TATGTAAGGCGTGGGTCA-3′ R:5′-TATGTAAGGCGTGGGTCA-3’	198 bp	XM_414406
*ACOX3*	F: 5’-ACATCTGGCTGTGCTCTATC-3′ R:5′-ACTCCCCGCTAGCTTTAC-3’	179 bp	XM_420814
*CPT1A*	F: 5’-AGACGGACACTGCAAAGGAG-3′ R:5′-AGCCCCTTCCCAAAAACA-3’	174 bp	NM_001012898
*CPT2*	F: 5’-GGGTCGTGTTGGGCTGTT-3′ R:5′-AAAGAGGTTTCTGGGCGTTC-3’	168 bp	XM_001234342
*DAK*	F:5’-AGAGGAGGAAGGAATTGACCTC-3′ R:5′-GTCGAAGACCACATGGCTGT-3’	272 bp	NM_001079500
*APOO*	F: 5’-CTGCCTTCTGCCTCAGGAAA-3′ R:5′-CAATGCTGATCCTGCAACGG-3’	162 bp	XM_015272548
*FUT9*	F: 5’-TGAAATGTGTAGCTGCGTGGA-3′ R:5′-AGACGTCTCCGAATTGCTTGT-3’	141 bp	NM_001079502
*GCNT1*	F: 5’-ACCAAGATACTGGAGGGCGA-3′ R:5′-CTCACTGCTGAGAGGTTCCA-3’	174 bp	XM_003643022
*B4GALT3*	F:5’-TCCTCCTGCACGATGTGAAC-3′ R:5′-TCGCCCCAGTATGTGTTTGG-3’	202 bp	XM_416564

qPCR analysis was performed after a reverse transcription reaction, as previously described [12]. cDNA was prepared with 2.0 μg of total RNA of each sample, in accordance with the manufacturer’s instructions. For qPCR, each PCR mixture with a volume of 25 μL contained 12.5 μL of 2 × iQ™ SYBR Green Supermix, 0.5 μL (10 mmol/L) of each primer, and 1 μL of cDNA. Mixtures were incubated in an iCycler iQ Real-time Detection system (Bio-Rad, Hercules, CA, USA) programmed to conduct 40 cycles (95 °C for 15 s and 65 °C for 35 s). Quantitation of the transcripts was performed using a standard curve with 10-fold serial dilutions of cDNA. A melting curve was constructed to ensure that only a single PCR product was amplified. Samples were assayed in triplicate with standard deviations of threshold cycle (CT) values not exceeding 0.5, and each experiment was repeated at least twice. Negative (without template) control reactions were performed for each sample.

### Gene ontology (GO) enrichment analysis and visualization

GO enrichment analysis was performed to identify the gene function classes and categories corresponding to the DEGs using the GOEAST software toolkit. The significance level for GO term enrichment was set at a false discovery rate (FDR) adjusted to less than 0.5 and a *P*-value of less than 0.05, by the Yekutieli method.

### Kyoto encyclopedia of genes and genomes (KEGG) pathway analysis

KEGG pathway [13, 14] information was also used in the analysis. A ProbeName for each category was first mapped to an NCBI Entrez gene ID according to the Agilent Chicken microarray annotation file, and then each was mapped to an appropriate KEGG gene ID according to the KEGG gene cross-reference file. Pathways that were significantly enriched with DEGs were identified using a hypergeometric test from the R package (*P* < 0.1, FDR-adjusted). Pathways with fewer than three known chicken genes were discarded.

### Statistical analyses

The significance of differences between groups was evaluated using Student’s *t*-test. *P* < 0.05 (*) or <0.01 (**) was considered significant. Data are presented as mean ± SEM.

## Results

### Fast-growing chickens had higher levels of TYE and fat deposition at hatching than did slow-growing chickens

**Fig. 1:**
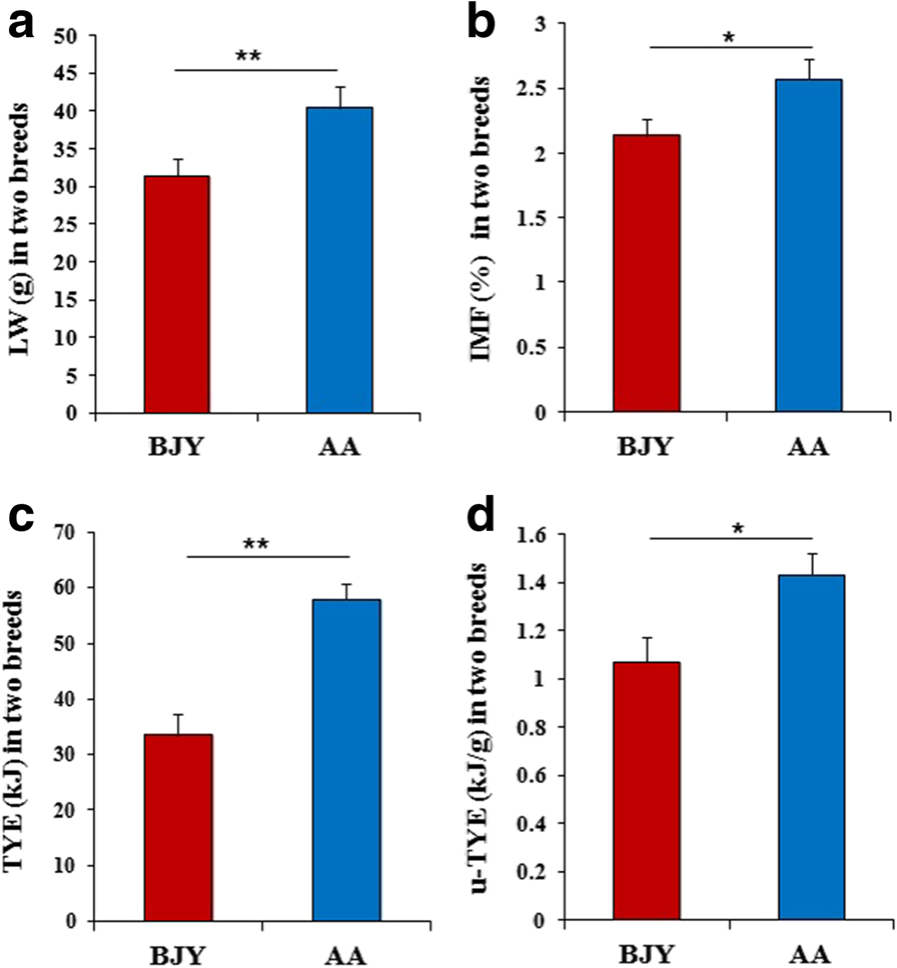
Summary of TYE, u-TYE, and IMF content in AA and BJY chickens on the day of hatching. Means within the same panel indicate significant differences between the two breeds (*P* < 0.01 or *P* < 0.05). Data are presented as mean ± SEM (*n* = 6)

Data on live weight (LW), IMF content, absolute TYE amount, and LW-specific TYE amount (u-TYE) in the two breeds are plotted in Fig. 1a–d. The content of IMF in the pectoralis major of AA chickens (2.57%) was significantly higher (*P* < 0.05) than that (2.14%) of BJY chickens. Similarly, the LW, TYE, and LW-specific u-TYE amounts were also significantly higher (*P* < 0.01, *P* < 0.01, *P* < 0.05) in AA chickens (40.46 g, 57.92 kJ, 1.43 kJ/g) than in BJY ones (31.39 g, 33.58 kJ, 1.07 kJ/g).

**Fig. 2:**
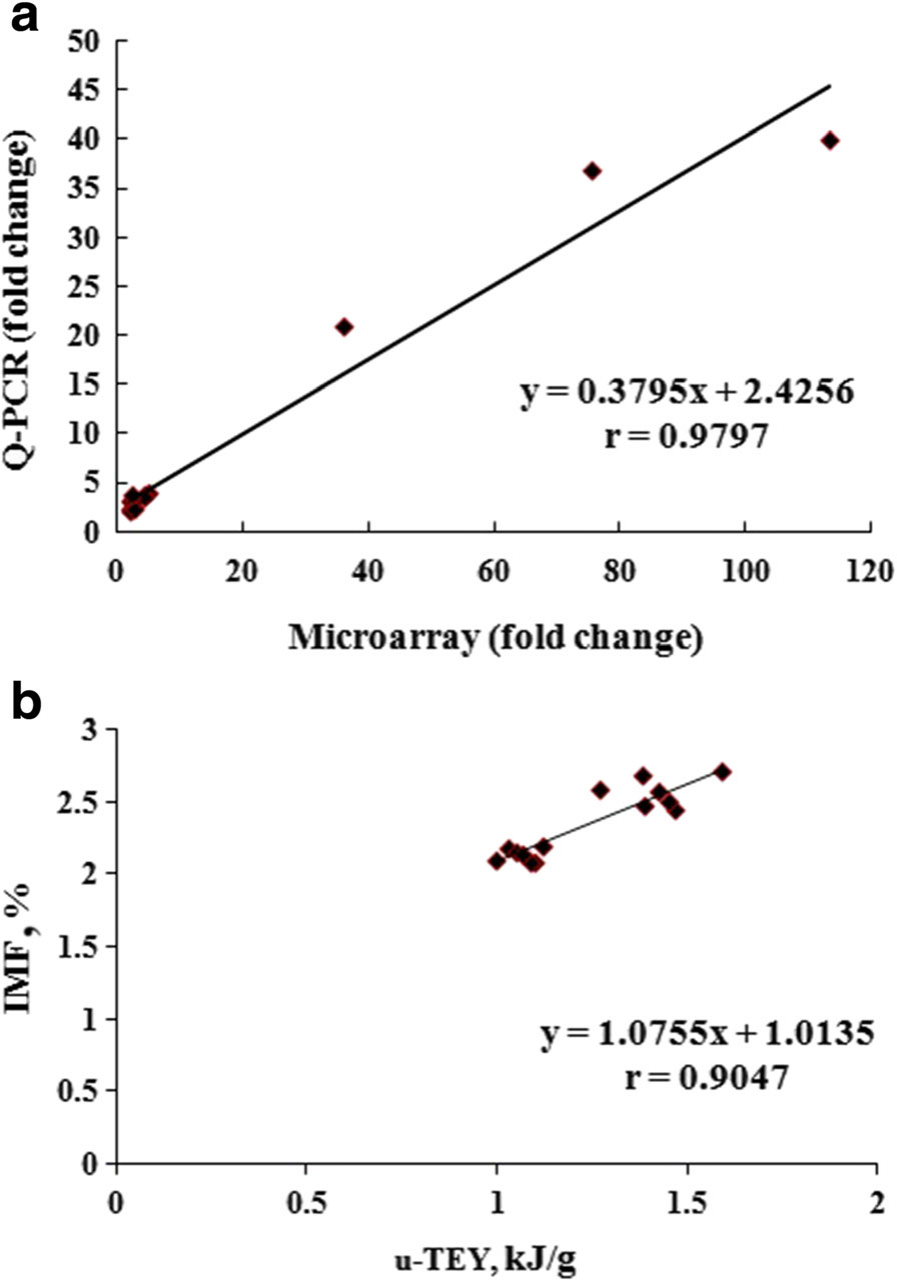
The correlation analysis by Spearman rank correlation in fast- (AA) and slow-growing (BJY) chickens. The high correlation coefficient (*r* = 0.9047) indicates that the IMF content correlated strongly with u-TYE in the two breeds (*n* = 12). The very high correlation coefficient (*r* = 0.9797) indicates that the qPCR fold changes of the two breeds correlated strongly with the microarray data (*n* = 15)

As shown in Fig. 2a, the correlation between IMF and u-TYE was *r* = 0.9047 (*P* < 0.01). There was more fat deposition in the pectoralis major of AA chickens than in BJY chickens on the day of hatching, which was suggested to have occurred because more energy had been supplied from the yolk sac in the former group.

### Higher expression of genes related to lipid biosynthesis in muscle of fast-growing than slow-growing chickens

Using an Agilent Chicken Gene microarray, a total of 787 known DEGs, 364 upregulated and 423 downregulated ones, were found in the pectoralis major of AA chickens at hatching, compared with their levels in BJY chickens (Additional file 1). Based on these 787 known DEGs, GO analysis was performed and GO terms enriched (*P* < 0.05) for biological processes were selected, as presented in Additional file 2. Based on GO-term analysis, 44 known DEGs related to lipid metabolism were screened. Compared with their levels in BJY chicken, 25 upregulated and 19 downregulated DEGs related to lipid metabolism were identified in AA chickens (Additional file 3), which are involved in many biological pathways: fatty acid biosynthesis, preadipocyte differentiation, triglyceride biosynthesis, and fatty acid degradation.

**Fig. 3:**
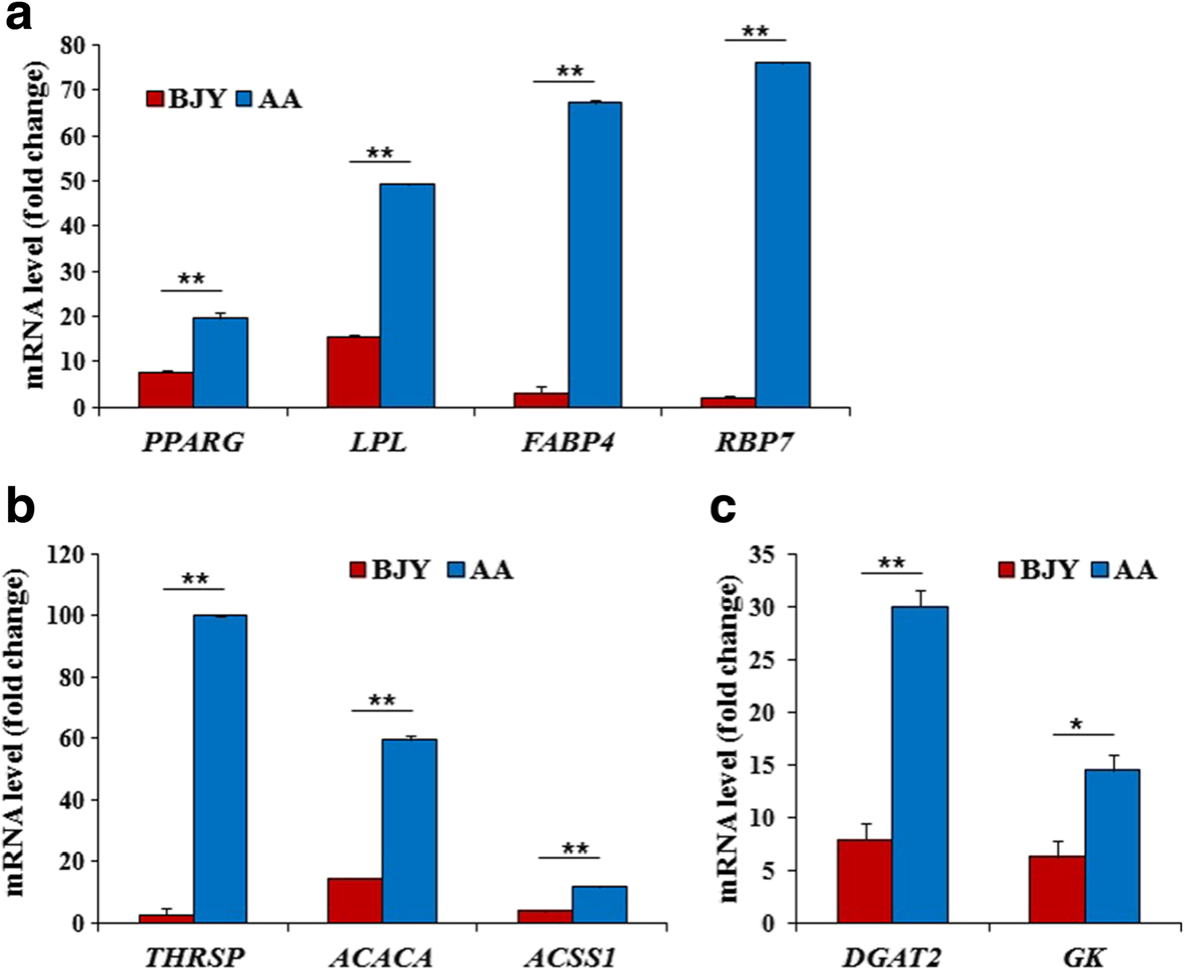
The expression levels of DEGs related to lipid biosynthesis determined by qPCR in fast- (AA) and slow-growing (BJY) chickens. These genes are all involved in fatty acid biosynthesis, preadipocyte differentiation, or triglyceride biosynthesis. All of these DEGs were significantly (*P* < 0.01 or *P* < 0.05) more highly expressed in AA chickens than in BJY chickens. Data are presented as mean ± SEM (*n* = 6)

From these 44 DEGs, 15 representative ones were selected to validate the microarray results by qPCR, and the correlation of the fold changes of the two breeds between these two sets of results (Fig. 2b) was *r* = 0.9797 (*P* < 0.01), indicating extremely strong correspondence for all 15 genes. It was also found that the relative expression of 9 of the 15 genes related to fatty acid biosynthesis (*THRSP*, *ACACA*, *ACSS1*) (Fig. 3a), preadipocyte differentiation (*PPARG*, *LPL*, *FABP4*, *RBP7*) (Fig. 3b), and triglyceride biosynthesis (*DGAT2*, *GK*) (Fig. 3c) was significantly upregulated (*P* < 0.05 or *P* < 0.01) in AA compared with the level in BJY chickens, consistent with the differences in lipid deposition. These results suggested that these genes are responsible for the greater IMF deposition in fast-growing chickens than in slow-growing ones.

### Lower expression of genes related to fatty acid degradation and glycometabolism in fast-growing than in slow-growing chickens

**Fig. 4:**
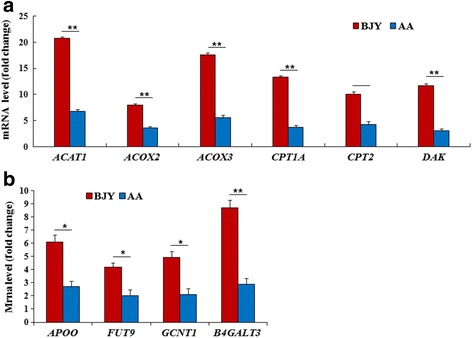
The expression levels of DEGs related to fatty acid degradation or glycometabolism determined by qPCR in fast- (AA) and slow-growing (BJY) chickens. These genes are all involved in fatty acid degradation or glycometabolism. Each of these DEGs was significantly (*P* < 0.01 or *P* < 0.05) downregulated in AA chickens compared with its level in BJY chickens. Data are presented as mean ± SEM (*n* = 6)

The expression levels of 10 genes (among 44 genes) related to fatty acid degradation (*ACAT1*, *CPT1A*, *CPT2*, *DAK*, *ACOX2*, *ACOX3*) and glycometabolism (*APOO*, *FUT9*, *GCNT1*, *B4GALT3*) were significantly lower (*P* < 0.05 or *P* < 0.01) in AA than in BJY chickens. Verification of these microarray results was obtained by qPCR, which showed that the expression of these 10 genes, related to fatty acid degradation (*ACAT1*, *CPT1A*, *CPT2*, *DAK*, *ACOX2*, *ACOX3*) (Fig. 4a) and glycometabolism (*APOO*, *FUT9*, *GCNT1*, *B4GALT3*) (Fig. 4b), was significantly lower (*P* < 0.05 or *P* < 0.01) in AA chickens than in BJY chickens.

The difference in expression level of these genes between the two breeds was consistent with these genes possibly contributing to greater deposition of IMF in the pectoralis major of AA chickens than that in BJY chickens at hatching.

### PPAR and other related signaling pathways regulate differences of expression in related DEGs between the two breeds at birth

A KEGG pathway analysis was performed on the 787 known DEGs to investigate the regulation of lipid metabolism in the pectoralis major at hatching. Seventeen KEGG pathways were identified in AA and BJY chickens (Additional file 4). Based on the 44 known DEGs related to lipid metabolism, another pathway analysis was performed and 7 KEGG pathways were identified in the two breeds (Additional file 5); then, five common enriched pathways (PPAR signaling, fatty acid metabolism, Hedgehog signaling, TGF-beta signaling, and cytokine–cytokine receptor interactions) were identified by the two KEGG pathway analysis methods.

## Discussion

Lipid is an essential energy source and cell membrane component in animals and, in chickens, is mainly deposited in abdominal, subcutaneous, and muscle adipose tissues. Although global gene expression research has been performed on abdominal adipose tissues [15–17], systematic research on the molecular regulation of IMF deposition in chicken pectoralis major at hatching has not. In this study, gene expression profiling was used to identify global DEGs and the pathways related to lipid metabolism in the breast muscle of fast- and slow-growing chickens at hatching.

### cDNA array analysis

RNA samples were pooled from individuals (*n* = 6) of each breed, as such a pooling strategy can dramatically improve accuracy when only one array is available for each biological condition [18]. Seventeen genes that are well known to be related to lipid metabolism were selected, and nearly 100 qPCR tests were performed to confirm the microarray results. Overall, 2.16% of the total DEGs (38.64% of the DEGs related to lipid metabolism) were verified as being present. As shown in Fig. 2b, the fold changes in gene expression strongly corresponded (*r* = 0.9797, *P* < 0.01) between the qPCR and microarray analyses. Despite the microarray analysis being performed once for each breed, the data exhibited high reliability and persuasive results were ensured because of the RNA pooling strategy and the high degree of verification.

### DEGs related to lipid metabolism in chicken pectoralis major at hatching

The yolk is the sole source of energy during the embryonic stages and at hatching. The remaining yolk energy supply upon hatching is absorbed and transported to tissues for deposition. The TYE and deposition of IMF in the pectoralis major in AA chickens were significantly higher (*P* < 0.01, *P* < 0.05) than those in BJY chickens at hatching, and were correlated in both breeds.

The energy remaining in birds at hatching is recovered from the yolk and stored (deposited) as IMF after transport, uptake, and re-esterification; a series of genes regulate these processes. To reveal the molecular regulation of IMF deposition in chicken pectoralis major at hatching, DEGs related to lipid metabolism were identified in the fast- and slow-growing chicken breeds.

These DEGs include *Spot 14* (encoded by *THRSP*) [19, 20], *ACACA* [21], and *ACSS1* [22, 23] play important roles in lipid metabolism by accelerating fatty acid biosynthesis. *PPARG*, *RBP7*, *LPL*, and *FABP4*, which positively regulate the process of preadipocyte differentiation [24, 25]. Similarly, *DGAT2* and *GK* promote esterification [26, 27]. All of these nine genes had significantly higher expression in the pectoralis major of AA chickens than in BJY chickens at hatching. Conversely, the expression of several genes was significantly lower in AA chickens than in BJY chickens. Among these, *ACAT1*, *ACOX2*, *ACOX3*, *CPT1A*, *CPT2*, and *DAK* positively regulate different steps of fatty acid oxidation [28–31], and *FUT9*, *GCNT1*, *APOO*, and *B4GALT3* all positively regulate energy use in maintaining metabolic balance between carbohydrates and lipids [32–36].

All of these differences in gene expression were either positively or negatively correlated with IMF content. This suggests that these genes play a role in regulating IMF deposition in the pectoralis major of chickens at hatching.

### Signaling pathways related to lipid metabolism in breast muscle at hatching

GO-term analysis was used to explore the function of the DEGs, and KEGG pathway analysis was used to explore the regulatory networks underlying IMF content. As expected, several well-known pathways relating to lipid metabolism were identified, including PPAR signaling, fatty acid metabolism, Hedgehog, TGF-beta, and cytokine–cytokine receptor interactions [37–40].

The PPAR signaling pathway is known to play an important role in regulating lipid metabolism [40, 41]. Many DEGs identified here are involved in PPAR signaling pathways, including *ACOX2*, *ACOX3*, *CPT1A*, *CPT2*, *CYP8B*, *DBI*, *FABP4*, *GK*, *LPL*, and *PPARG*. Several DEGs, including those of the *BMP* family and receptors (*BMP2*, *BMP5*, *BMP7*, *BMPR1B*), *LEPR*, and *SHH* participate in the Hedgehog, TGF-beta, and cytokine–cytokine receptor interaction signaling pathways. The expression levels of *bone morphogenetic proteins 2* (*BMP2*), *bone morphogenetic proteins 5* (*BMP5*), and *bone morphogenetic proteins 7* (*BMP7*), and *BMP receptor 1B* (*BMPR1B*), *sonic Hedgehog homolog* (*SHH*), and *leptin receptor* (*LEPR*) were all significantly different between the two breeds (Additional file 3). Previous studies have shown that Hedgehog, TGF-beta, and cytokine–cytokine receptor interaction signaling pathways can regulate lipid metabolism [37–39] through the PPAR signaling pathway. This is consistent with the present results from KEGG pathway analysis. The cytokine–cytokine receptor interaction signaling pathway can regulate cell differentiation through the *LEPR* and *TGF* families (*TGF-beta* and *BMP*). Therefore, the cytokine–cytokine receptor interaction signaling pathway may share a similar role to the Hedgehog and TGF-beta signaling pathways as an upstream regulator of the PPAR signaling pathway in lipid metabolism.

**Fig. 5:**
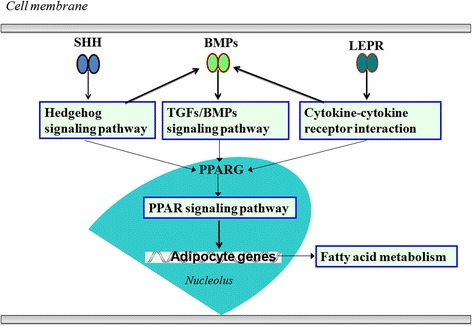
Lipid metabolism regulatory network proposed for the breast of female chickens at hatching, based on significant DEGs and KEGG pathway analysis. The network involves Hedgehog, TGF-beta, and cytokine–cytokine receptor interaction signaling pathways, through *SHH*, *BMP*, and *LEPR* molecular interactions. These three pathways potentially regulate lipid metabolism via the PPAR signaling pathway

Several DEGs, including the *BMP* family and its receptors, participated in more than one of the Hedgehog, TGF-beta, and cytokine–cytokine receptor interaction signaling pathways in the present study, so it is suggested that all of the Hedgehog, TGF-beta, and cytokine–cytokine receptor interaction signaling pathways play roles in the upstream regulation of the PPAR signaling pathway in lipid metabolism. These results suggest that these pathways form a network, along with others related to lipid metabolism, to influence IMF deposition in the chicken pectoralis major at hatching (Fig. 5). The KEGG pathway analysis suggests that lipid metabolism in chicken pectoralis major at hatching is regulated both directly by genes encoding participating enzymes and indirectly via signaling pathways.

## Conclusion

In summary, residual sources of energy from the yolk sac are transported to be deposited as IMF in chickens at hatching. Genes and pathways related to lipid metabolism (such as PPAR, Hedgehog, TGF-beta, and cytokine–cytokine receptor interaction signaling pathways) account for greater IMF deposition in the pectoralis major of fast-growing chickens (AA) than that in a slow-growing breed (BJY)*.* These findings provide new insights into the molecular mechanisms underlying lipid metabolism in chickens at hatching.

## Additional files

Additional file 1: Annotation and changing of 787 DEGs in pectoralis major of AA and BJY chickens at hatching. (XLS 207 kb)

Additional file 2: The enriched GO terms among the 787 DEGs in both AA and BYJ chickens. (XLS 64 kb)

Additional file 3: A total of 44 known DEGs related to lipid metabolism in AA and BJY chickens. (XLS 59 kb)

Additional file 4: The enriched KEGG pathways based on 787 known DEGs in AA and BJY chickens. (XLS 57 kb)

Additional file 5: The enriched KEGG pathways based on 44 known DEGs related to lipid metabolism in AA and BJY chickens. (XLS 52 kb)

## Abbreviations

AA: Arbor acres chicken
ACACA: Acetyl-coenzyme A carboxylase alpha
ACAT1: Acetyl-coenzyme A acetyltransferase 1
ACOX2: Acyl-coenzyme A oxidase 2
ACOX3: Acyl-coenzyme A oxidase 3
ACSS1: Acyl-CoA synthetase short-chain family member 1
APOO: Apolipoprotein O
B4GALT3: UDP-Gal:betaGlcNAc beta 1,4- galactosyl transferase, polypeptide 3
BJY: Beijing you chicken
CPT1A: Carnitine palmitoyltransferase IA
CPT2: Carnitine palmitoyltransferase II
DAK: Dihydroxyacetone kinase 2 homolog
DEGs: Differentially expressed genes
DGAT2: Diacylglycerol O-acyltransferase homolog 2
FABP4: Fatty acid binding protein 4
FDR: False discovery rate
FUT9: Fucosyltransferase 9
GCNT1: N-glucosaminyl (N-acetyl) transferase 1
GK: Glycerol kinase
GO: Gene ontology
KEGG: Kyoto encyclopedia of genes and genomes
LPL: Lipoprotein lipase
LW: Live weight
PPARG: Peroxisome proliferator-activated receptor gamma
qPCR: Quantitative real time PCR
RBP7: Retinol binding protein 7
THRSP: Thyroid hormone responsive
TYE: Total yolk energy
u-TYE: LW-specific TYE amount
